# Comparison of mHTT Antibodies in Huntington’s Disease Mouse Models Reveal Specific Binding Profiles and Steady-State Ubiquitin Levels with Disease Development

**DOI:** 10.1371/journal.pone.0155834

**Published:** 2016-05-19

**Authors:** Zubeyde Bayram-Weston, Lesley Jones, Stephen B. Dunnett, Simon P. Brooks

**Affiliations:** 1 Division of Neuroscience, School of Biosciences, Cardiff University, Cardiff, Wales, United Kingdom; 2 Institute of Psychological Medicine and Clinical Neurosciences, Cardiff University School of Medicine, Cardiff, Wales, United Kingdom; Grenoble Institut des Neurosciences, Universite Grenoble Alpes, FRANCE

## Abstract

Huntington’s disease (HD) cellular pathology is characterised by the aggregation of mutant huntingtin (mHTT) protein into inclusion bodies. The present paper compared the sensitivity of five widely used mHTT antibodies (S830; MW8; EM48; 1C2; ubiquitin) against mice from five commonly used HD mouse models (R6/1; YAC128; HdhQ92; B6 HdhQ150; B6 x129/Ola HdhQ150) at two ages to determine: the most sensitive antibodies for each model; whether mHTT antibody binding differed depending on aggregation stage (diffuse versus frank inclusion); the role of ubiquitin during aggregation as the ubiquitin proteosome system has been implicated in disease development. The models demonstrated unique profiles of antibody binding even when the models varied only by background strain (HdhQ150). MW8 was highly sensitive for detecting frank inclusions in all lines whereas EM48, ubiquitin and 1C2 demonstrated consistent staining in all models irrespective of age or form of mHTT. MW8 and S830 were the most sensitive antibodies with 1C2 the least. Ubiquitin levels were stable for each model regardless of age. Ubiquitin was particularly sensitive in young YAC128 mice that demonstrate an absence of inclusions until ~12 months of age suggesting high affinity to mHTT in its diffuse form. The data indicate that generalisations across models regarding the quantification of aggregations may not be valid and that mHTT antibody binding is unique to the mouse model and sensitive to changes in inclusion development.

## Introduction

Huntington's disease (HD) is an inherited progressive neurodegenerative disorder caused by a CAG repeat expansion in the first exon of *HTT*. *HTT* encodes the huntingtin protein (HTT) and the CAG repeat tract gives rise to a polyglutamine tract in the protein [1]. The onset of the disease is generally in mid-life with death occurring 15–20 years later. HD is characterized by characteristic motor and cognitive impairments and variable psychiatric symptoms, associated with selective cellular dysfunction and neuronal loss first within the caudate nucleus and later across the whole brain [2]. Neuronal intra-nuclear inclusions (NIIs) containing mutant HTT (mHTT) have been reported in patients [3–8] and model systems that carry a *HTT* polyglutamine mutation [9–17]. The precise role that the aggregation process and HTT-positive inclusions play in HD neuropathology remains unclear [6, 12, 18–29], but their presence in either the early aggregation diffuse state or as frank NIIs are predictors of cell death [22, 28, 30] and are at the very least, markers of regional neuropathology.

There are several mHTT antibodies that immunolabel mHTT. Within HD mouse models the antibodies bind to different regions of the mHTT protein. Some have affinity specifically for the expanded polyglutamine [31, 32] whilst others bind to the N—or C -terminal of the exon 1 region [6, 33, 34]. The epitope specificity of the antibodies can characterise the inclusions by showing the availability of different epitopes and therefore the potential conformational changes that mHTT undergoes during the development of inclusion formations [35]. The ubiquitin antibody has also been used to detect mHTT as it is sequestered during the aggregation process.

The ubiquitin tagging of proteins, including those that are misfolded, marks them for degradation via the ubiquitin-proteasome system (UPS) [36, 37]. Altered ubiquitination has been proposed as a pathological mechanism in HD cell death [38–40] and inhibition of the proteasome in HD cell produced an increases in mHTT aggregation [38, 41]. A recent study [26] suggested that ubiquitination of NIIs is a late stage event in the development of the inclusion and therefore of little pathological relevance, in contrast to an earlier report that ubiquitin staining was already present in NIIs from their inception/early stages of formation [42]. Both of these studies were limited by their use of a single antibody and a single HD mouse line (R6/2) making an objective interpretation of ubiquitin binding within and beyond this single mouse line difficult. Another recent study [43] found ubiquitin activity that increased with age in both cortex and striatum of the 84Q transgenic HD mouse line, and although the authors claim the increases appear prior to the onset of HD in the mice, the ubiquitin increase was consistent with the appearance and progression of EM48 mHTT staining, suggestive of close and ongoing interactions between the aggregation and ubiquitination processes. At present it is not clear how ubiquitin interacts with mHTT and whether this is an early or late stage event in the development of mHTT inclusion pathology.

As part of a larger programme of work characterising HD mouse lines, we sought to characterise the ability of the most commonly used antibodies for the detection of mHTT (S830, MW8, EM48, 1C2) within striatal tissue of 4 congenic (C57BL6/j hereafter referred to as B6) HD mouse lines (R6/1, HdhQ92, YAC128, HdhQ150). Since there is a general movement to breed genetically modified mouse lines to common background strains to overcome background strain confounds particularly in behavioural phenotypes, we included a second variant of the HdhQ150 mouse line which was maintained on its original background strain (129/Ola X C57BL/6 background) to determine the impact of background on aggregation neuropathology. Our previous studies [44–47] demonstrated the predominance of early disease diffuse mHTT staining to the staining of frank NIIs with age in these mouse lines, and thereby provided age groups for the present study for the comparison of antibody sensitivity to the different forms of mHTT. We also sought to determine whether ubiquitin tagging of the mHTT in HD mouse models is primarily related to mHTT in its diffuse state or associated in the development of frank inclusion pathology as both notions have been suggested to be of importance to the development of HD neuropathology [26, 42].

## Materials and Methods

### Animals

All experiments were conducted under UK licence and in accordance with the UK Animals (Scientific Procedures) Act 1986 and Cardiff Biological Standards Committee. All mice were killed by lethal IP injection of Ethatal conforming to UK and European legislation.

The mice used were congenic to the C57BL/6 background with the exception of the HdhQ150 mouse on the original 129/Ola X C57BL/6 background (see Table 1). For each line, tissue samples were taken from 5–8 mutant gene carriers at 8 and 18 months of age to provide “young” and “old” data points for each of the lines with the exception of the R6/1 mice that have a shortened life span where tissue was taken at 4 and 7 months of age as being a proportional life span representation of the other lines.

**Table 1:** Characteristics of the mouse lines used in the present study.

Line	CAG number (mean)	Construct
R6/1 (B6)	124	Human exon 1 (15)
HdhQ92 (B6)	90	Hdh/HD exon 1 with human polyglutamine and flanking domains *Htt* (48)
YAC128 (B6)	126	Full Human *HTT* in genomic context (11)
HdhQ150 (B6)	146	Mouse *Htt* with human polyglutamine length (17)
HdhQ150 (B6 X 129Ola)	151	Mouse *Htt* with human polyglutamine length (17)

All but one of the mouse lines was bred on a C57BL6/j (shortened to B6) background and all varied by CAG repeat length. The inserted genetic construct was of either a fragment (R6/1), artificial chromosome (YAC128), or knock-in (HdhQ92 and HdhQ150).

The R6/1 mice [15] were originally obtained from Jackson Laboratory (Maine, USA) and backcrossed to the pure B6 background for >10 generations. The YAC128 mice [11] were kindly supplied by the Hayden laboratory (University of British Columbia, Vancouver, Canada) and were backcrossed on to a B6 background for >10 generations. The HdhQ92 mice [48] were bred in-house but originated from stock animals (Jackson Laboratories, Maine, USA), and backcrossed onto the B6 background over a minimum of six generations. The “original” HdhQ150 mouse line[17] was bred in-house on the original mixed 129/Ola X B6 background strain and maintained on an F1 cross with a second line bred and maintained in house on a pure B6 background.

All mice were genotyped commercially (Laragen Inc., Culver City, USA) by the tail tip samples as previously described [44–47]. Prior to sacrifice the mice were housed in mixed genotype single-sex cages under standard animal laboratory conditions. Animals were kept on a 12 hour lights on/off light-dark cycle (lights on 07:00h) with *ad libitum* access to food and water and an ambient room temperature of 21±1°C.

### Histology

#### Tissue preparation

All mice and tissues were treated in the same way throughout the tissue handling process except where described. The mice were anaesthetized by intraperitoneal injection of 0.2 ml of Euthetal (Merial, Essex, UK) and then perfused intracardially with phosphate-buffered saline (PBS, pH 7.4) for 3 min. Followed by 4% paraformaldehyde (PFA) (Fisher Scientific, Loughborough, UK) in a 0.1M PBS solution, pH 7.4, for a further 5 min. The brains were carefully removed, post fixed in 4% PFA for 4 h, and then transferred to 25% sucrose in PBS for 24 h or until they sunk to the bottom of the container. The coronal sections (40 μm) of the brain were cut in series of 1:12 using a freezing sledge microtome (Leitz Bright Series 8000, Germany) and were cryoprotected by immersion in antifreeze solution. Prior to immunohistochemistry tissue sections for each antibody were placed in (pH 7.4) TRIS Buffered Saline (TBS), and washed twice for 5 min. Sections for the ubiquitin, S830, EM48 and MW8 antibodies were pre-treated with an antigen retrieval method by incubation in citrate buffer (pH 6) for 20 min at 95C and for the 1C2 antibody which was insensitive to citric acid treatment, a 90% formic acid solution was used for 5 min at room temperature. Methods were adapted from in house-procedures and those previously described [10, 49]. Staining with all of the primary antibodies was optimised based on previous reports prior to the study to determine the optimal parameters for visualisation of mHTT staining.

#### mHTT Primary antibody immunohistochemistry

Endogenous peroxidise activity was inhibited by incubation in methanol containing 3% H_2_O_2_ (VWR International, UK) for 5 min. Non-specific binding sites were blocked with 3% horse serum in TBS for 1 h, and the sections were incubated with ubiquitin (1:1000:1μl in 1ml TXTBS containing 1% horse serum), S830 (1:20000: 1μl in 20ml TXTBS containing 1% horse serum), EM48 (1:500: 0.5μl in 1ml TXTBS containing 1% horse serum), MW8 (1:1000: 1μl in 1ml TXTBS containing 1% horse serum), or 1C2 (1:4000: 0.25μl in 1ml TXTBS containing 1% horse serum) antibodies overnight (~16 hours) at room temperature (21°± 2°C). After several washes in TBS, the sections were incubated with a horse anti-goat secondary antibody for S830 or horse anti-mouse secondary antibody (diluted 1:200, Vector Laboratories, Burlingame, CA, USA) for MW8, EM48, ubiquitin and 1C2 for 2 h at 21° ± 2°C. After several washes in TBS, the sections were incubated with a biotin-streptavidin kit according to the manufacturer’s instructions (Vector Laboratories). After which, the sections were rinsed in TBS. For each antibody the peroxidase activity was visualized with 3,3’-diaminobenzidine (DAB: Sigma-Aldrich, Poole, UK), and the sections mounted on gelatine-coated slides, prior to dehydration and cover-slipping (see Table 2 for details).

**Table 2:** Summary table of the primary antibodies used in the present study.

Antibody	Type	Antigenretrieval	Source	Binding locus
Ubiquitin[1:1000]	Mouse (mono)	Citric acid	Invitrogen UK, (cat: 13.1600)	Ubiquitin polypeptide (50)
S830[1:20000]	Sheep (poly)	Citric acid	Bates lab. Kings College London	mHTT: N-terminal exon 1 to 53Q (32)
EM48[1:500]	mouse (mono)	Citric acid	Millipore UK, (cat: MAB5374)	mHTT: N-terminal (AA1-212) of exon-1 and 82-150Q (16)
MW8[1:1000]	mouse (mono)	Citric acid	see “*” below	mHTT: C-Terminal 8AA (AEEPLHRP) sequence of exon1 (34)
1C2[1:4000]	mouse (mono)	Formic acid	Millipore UK, (cat: MAB1574)	polyglutamine stretches of >38Q (31)

The concentration used for each antibody is stated in square brackets beneath the antibody name, and mono/polyclonal nature and antibody specifies is summarised under the “Type” heading. All antibodies except 1C2 used citric acid antigen retrieval. Where the antibodies were obtained is listed under the “Source” heading (*MW8 was developed by Patterson and colleagues[34], was obtained from the Development Studies Hybridoma Bank, created by NICHD of the NIH and maintained at the University of Iowa, Department of Biology, Iowa City, IA52242), and the specific binding epitope of the antibodies is listed under the heading “Binding locus”. S830 was kindly provided by Prof Bates (Kings College London).

It should be noted that the presence of the ubiquitin polypeptide is assessed but we present no evidence that its presence is due to sequestration into the mHTT aggregate although this is widely accepted (see above). Consequently, ubiquitin staining is described as diffuse or concentrated as an indicator that it may be associated with diffuse or aggregated mHTT staining respectively.

#### Stereology

Two dimensional stereology was performed on an Olympus BX50 microscope (Olympus Optical Co. Tokyo, Japan) with PC-based image analysis software (Olympus C.A.S.T. grid system v1.6.). Cell counts were performed on a 1:6 series of S830-stained sections and 1:12 series of EM48, MW8, 1C2 and ubiquitin-stained sections throughout the entire left striatum of each mouse. Briefly, the striatum was outlined under a 4× objective lens and the enclosed area was calculated by the C.A.S.T grid software. Sections within a defined volume of the striatum were then sampled at random and cells were counted under a 100× objective lens using a 265 μm^2^ 2D optical dissector counting frame. The total number of affected cells in the structure per section was calculated using the Abercrombie correction[50]:

C=Σc × (ΣA/ Σa) × f

**C:** The total number of cells; **∑c:** The total number of cells counted; **∑A:** The sum of all the inclusion areas; **∑a:** The sum of all the sample area; **f:** The frequency of sectioning.

Affected cells were identified and the total numbers estimated in terms of positive immunoreactive-labelling; affected cells were further categorized in terms of whether they expressed either diffuse nuclear staining alone or exhibited frank NIIs with or without additional diffuse nuclear staining.

#### Statistical analyses

Initially, within strain 2-way (Antibody x Age) analyses of variance (ANOVA) were used to determine antibody binding differences in the detection of mHTT diffuse or inclusion cellular pathology within the striatum. With significance, post-hoc analysis was undertaken with pairwise analysis using Tukey HSD tests and for age-related analysis independent t-tests were used where appropriate. As there were many possible interactions available for analysis, selected main effects and interaction comparisons are reported, and post-hoc significances are simply marked by lowest significance level (in parenthesis) amongst the comparator groups. All statistical analyses were performed using the Genstat statistical package (v13.2; VSN International, Hemel Hempstead, UK) with significance taken at the 0.05 level.

## Results

### R6/1 (Fig 1 and photomicrographs A-E Figs 2 and 3)

**Fig 1:**
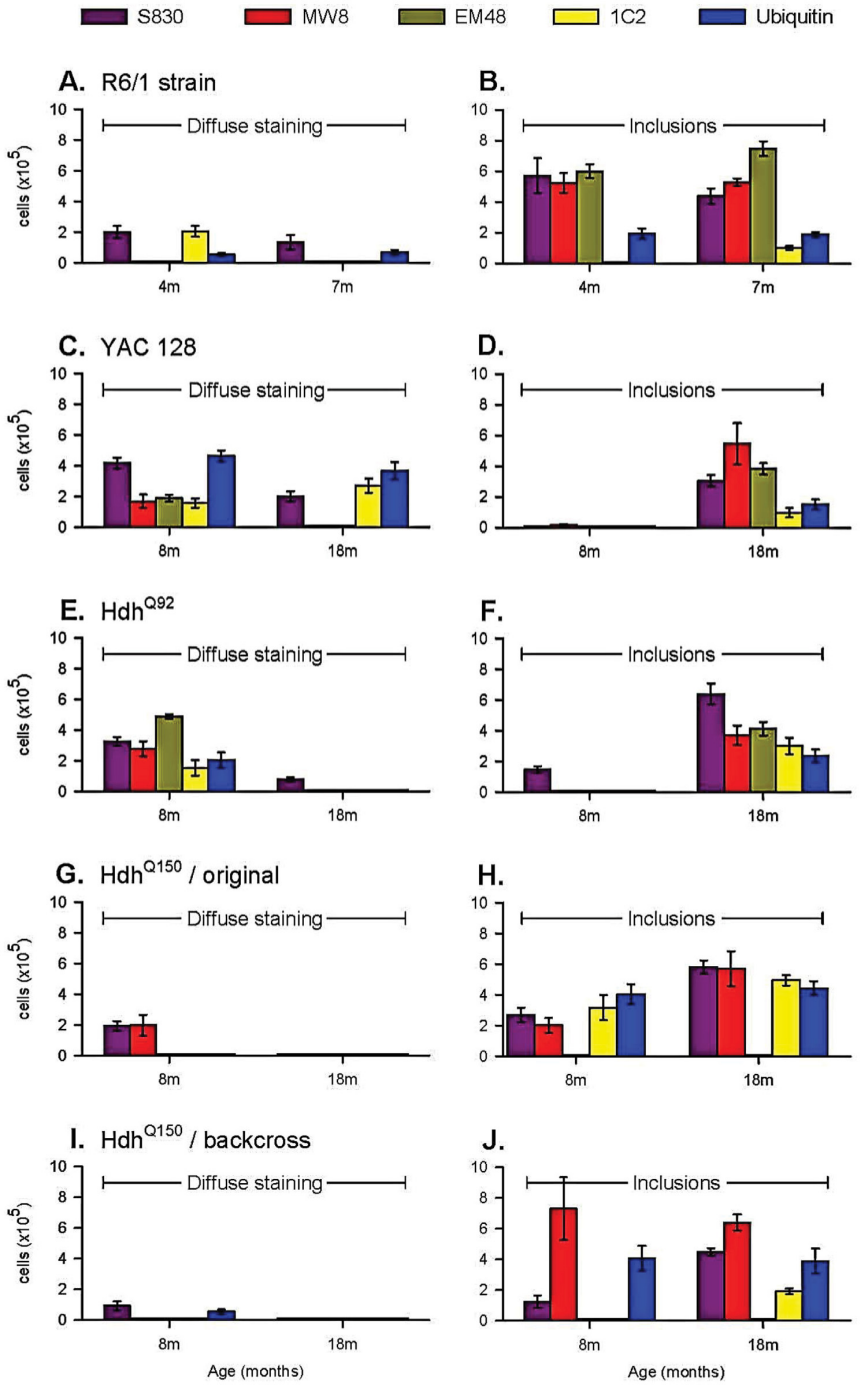
mHTT antibody binding in young and old animals from 5 HD mouse models. R6/1 mice demonstrate some diffuse staining (A) but high levels of frank inclusions from 4 months of age (B). In this mouse line EM48 was overall the most sensitive antibody, with 1C2 and ubiquitin the least. In young YAC128 mice that demonstrate only diffuse staining (C,D), ubiquitin and S830 were the most effective antibodies. As the mice aged MW8 became the most sensitive antibody. In the HdhQ92 mice, EM48 was the most effective antibody in the young animals (E) with S830 the most effective in the old (F) where the other four antibodies demonstrated a consistent level of mHTT detection. For the HdhQ150 strains, both lines were insensitive to EM48 and demonstrated relatively little binding of diffuse staining per se (original line G,H; B6 backcross I,J) with the antibodies detecting inclusions at high levels even at a young age. The original HdhQ150 line demonstrated consistent levels of detection with the four usable antibodies including 1C2, which was ineffective in the B6 line. MW8 was the most sensitive antibody in the B6 line. Significance markers omitted for clarity (See S1 File for raw data).

**Fig 2:**
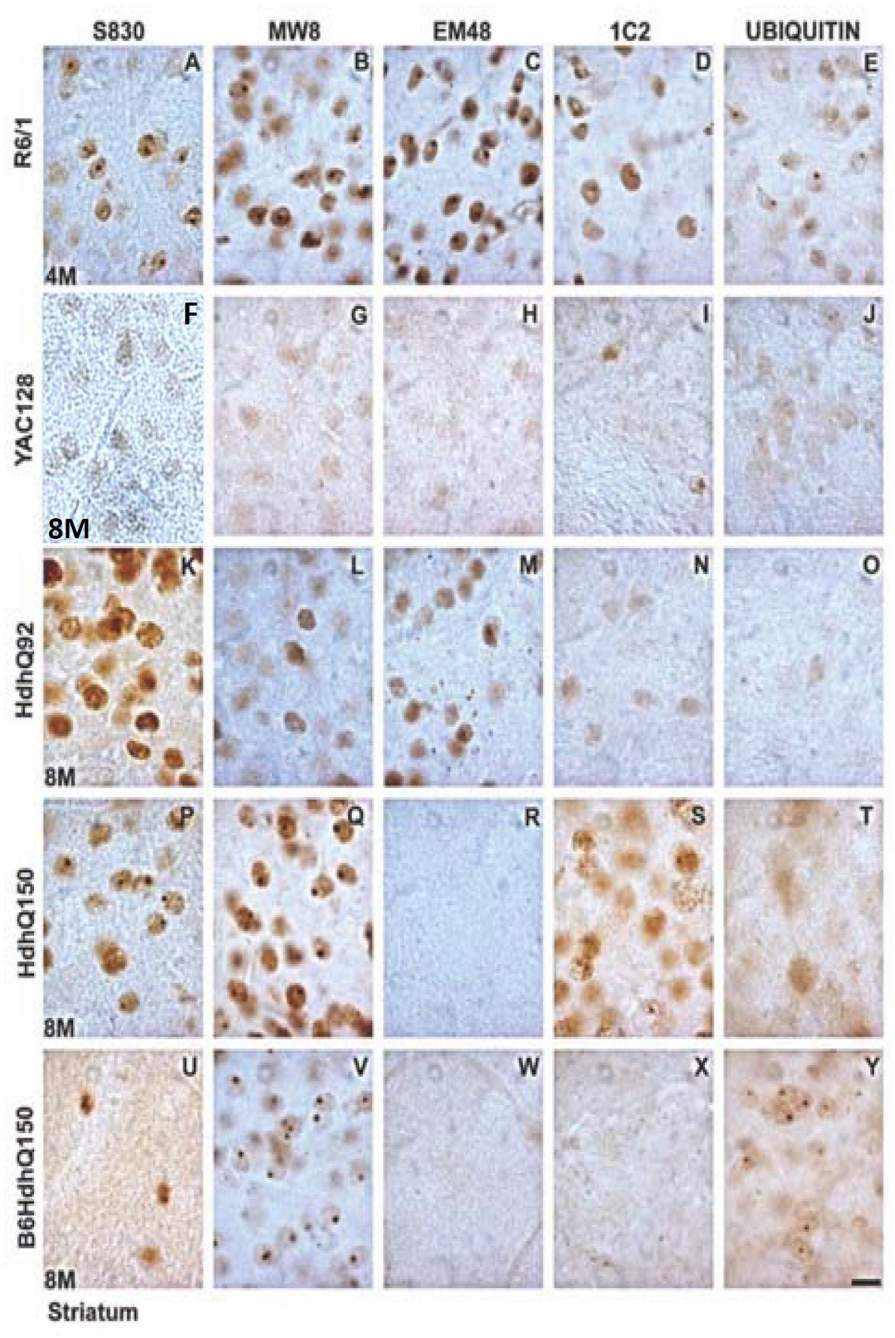
High power light microscopy images of mHTT antibody staining of striatal aggregation pathology in early disease HD mice. The R6/1 mice demonstrated NIIs with all antibodies (A,F,K,P,U), whereas the YAC128 mice demonstrate no NIIs but faint diffuse staining with all antibodies (G,L,Q,V) except S830 (B). The antibodies also failed to detect NIIs in the HdhQ92 line at this age (Photomicrographs C,H,M,R,W) but did demonstrate diffuse staining with all. Both HdhQ150 lines were insensitive to EM48 (N,O) with the original HdhQ150 line also being insensitive to 1C2 (X), but the Photomicrographs for S830 (D), MW8 (I) and ubiquitin (X) in the original line demonstrated good sensitivity for NIIs, as they did for the B6 variant (Photomicrographs E,J, Y respectively). Scale bar = 10 μm.

**Fig 3:**
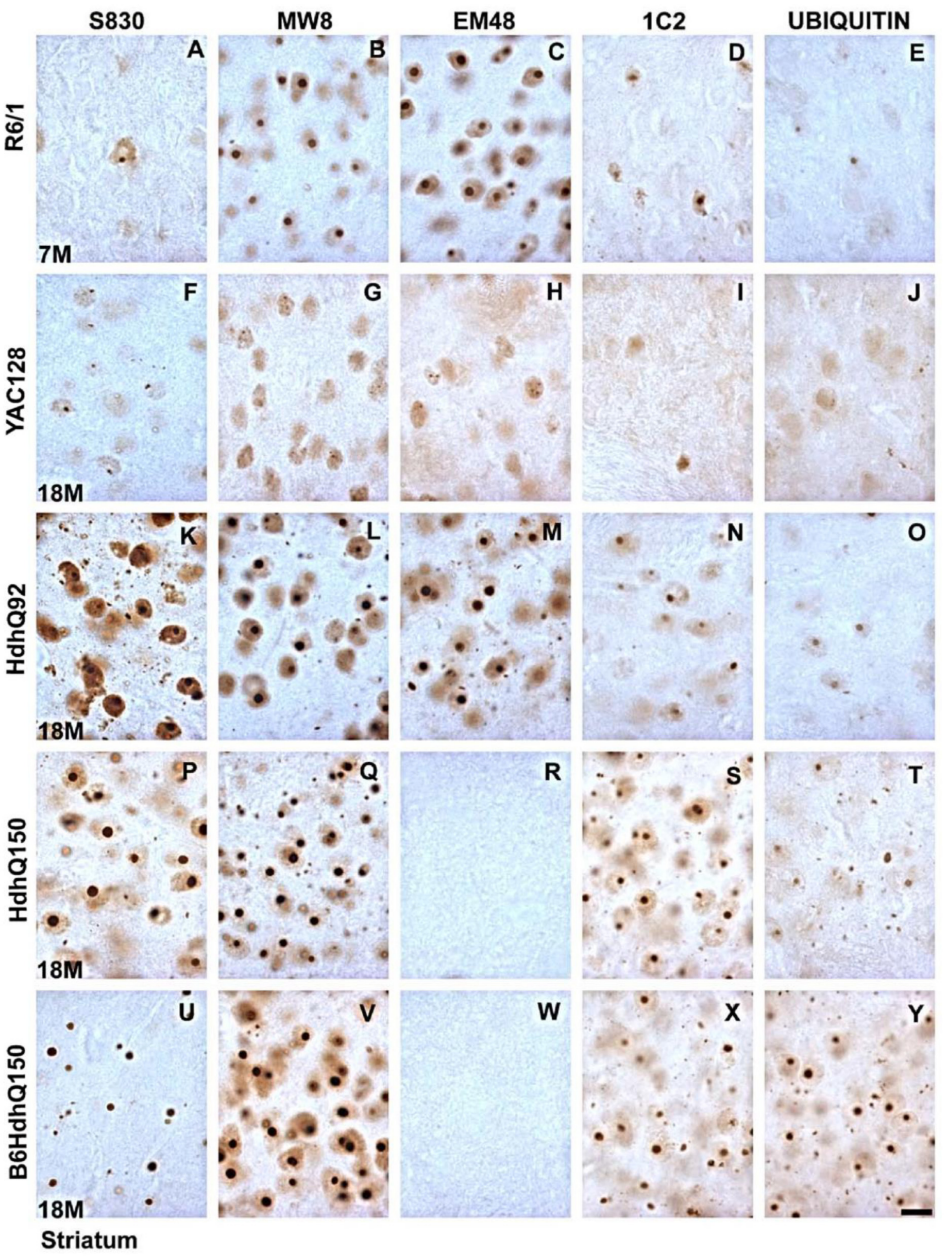
High power light microscopy images of mHTT antibody staining of striatal aggregation pathology in later disease HD mice. The R6/1, YAC128 and HdhQ92 mice were sensitive to each of the antibodies (R6/1 Photomicrographs A,F,K,P,U; YAC128 Photomicrographs B,G,L,Q,V; HdhQ92 Photomicrographs C,H,M,R,W), whereas both HdhQ150 lines where sensitive to all but EM48 (B6 HdhQ150 Photomicrographs E,J,O,T,Y; original HdhQ150 Photomicrographs D,I,N,S,X). Scale bar = 10 μm.

In the R6/1 mouse the level of diffuse staining changed differentially with age and antibody (Fig 1A: Age x Antibody, F_4,31_ = 7.15, p<0.001). Staining for frank inclusions also varied by antibody used (Fig 1B: Antibody: F_4,31_ = 48.49, p<0.01) with the relative sensitivity of the antibodies not changing with age (Age x Antibody, F_4,31_ = 2.18, n.s.), presumably as the disease is well advanced in these animals by 4m. Overall the most sensitive antibody in the R6/1 mouse was EM48 (p<0.05) that detected inclusions in high numbers but was not sensitive for diffuse staining. MW8 and S830 also detected good numbers of inclusions, but whereas MW8 also failed to detect diffuse mHTT consistent with the EM48 profile, S830 detected diffuse mHTT binding in both 4m old and 7m old animals. The 1C2 antibody demonstrated preferential binding to mHTT in the diffuse form in the 4m old animals and found relatively few frank inclusions in 7m old mice despite the high numbers detected by other antibodies. Ubiquitin staining was generally weak but stable at both ages in the R61 mice.

### YAC128 (Fig 1 and photomicrographs F-J of Figs 2 and 3)

The YAC128 mice provided the most unique aggregation profile of the lines in the present study. Striatal inclusion formations appeared late in the life of the mouse, despite the presence of diffuse mHTT staining at an early age. The cellular development of diffuse striatal mHTT staining in the absence of inclusions was differentially sensitive to the different antibodies (Fig 1C: Antibody x Age, F_4,34_ = 14.02, p<0.001), as was the development of frank inclusions (Fig 1D: Antibody x Age, F_4,36_ = 6.76, p<0.001).

In the 8m old YAC128 mice ubiquitin was the most effective antibody compared against all others except S830 (p<0.05). No antibody detected frank inclusions at 8m. The ubiquitin staining remained high for the diffusely stained cells in the 18m animals and demonstrated greater diffuse than punctate staining (p<0.01), suggesting that it was less able to detect frank inclusions in the same animals despite their presence in high numbers being detected by other antibodies. The 1C2 antibody demonstrated a similar propensity to detect diffuse staining over frank inclusions in the 18m old mice (p<0.01). In contrast, the sensitivity of MW8 increased markedly with age when mHTT became aggregated into inclusions (p<0.01). MW8, S830 and EM48 were the most sensitive antibodies for detecting inclusions (p<0.05 against 1C2 and the presence of ubiquitin). In the 18m old mice EM48 and MW8 failed to detect mHTT in cells that did not contain an inclusion.

### HdhQ92 (Fig 1 and photomicrographs K-O of Figs 2 and 3)

In the HdhQ92 mice the number of cells demonstrating diffuse staining in the absence of inclusions fell with age (Fig 1E: Antibody x Age, F_4,33_ = 13.01, p<0.001) with a concomitant increase in inclusions (Fig 1F: Antibody x Age, F_4,34_ = 4.01, p<0.01).

In the 8m old mice, post-hoc analysis revealed that of the antibodies used EM48 detected most mHTT (p<0.05). At 8m of age this was in the diffuse form but interestingly whilst this level of detection did not change with age, at 18m EM48 detected only inclusions. This general pattern was maintained for each of the antibodies although S830 was able to detect a modest number of inclusions at 8m. In the 18m old mice, post-hoc analysis revealed that S830 was the most sensitive antibody overall (p<0.05) for detecting inclusions and was the only one that was able to detect diffuse mHTT at this age. MW8, EM48, 1C2 and ubiquitin demonstrated comparable levels of diffuse staining at 8m of age and inclusions at 18 months of age.

### Original (B6 x 129/Ola) HdhQ150 (Fig 1 and photomicrographs P-T of Figs 2 and 3)

The lack of binding with the EM48 antibody in this line and the backcross B6 HdhQ150 line was expected and occurs due to the mismatch in human binding specificity of the antibody and the restricted human component of the inserted construct (CAG repeats only) of these models.

There were significant differences in the extent of detection of both diffuse staining (Fig 1G: Antibody x Age: F_4,29_ = 10.62, p<0.001) and inclusion detection (Fig 1H:Antibody x Age: F_4,29_ = 4.43, p<0.01) between the antibodies in the (B6 x129/Ola) HdhQ150 line.

All antibodies (except EM48) detected inclusions at 4m of age but only S830 and MW8 also detected cells with diffuse mHTT in the absence inclusions at this age. In the 8m old mice all of the antibodies were comparably sensitive at detecting inclusions. With advancing disease cells with diffuse mHTT and no inclusions became undetectable at 18m of age for all antibodies. In contrast all antibodies (except EM48) detected high levels of inclusions to comparable levels. Of interest in this mouse line was that whereas the detection of inclusions by S830, MW8 and 1C2 increased significantly with age (p<0.05), but the ubiquitin antibody maintained a constant level of staining across the age groups.

### B6 HdhQ150 (Fig 1 and photomicrographs U-Y of Figs 2 and 3)

As with all of the other mouse lines the detection of diffuse mHTT by the different antibodies across the two age groups differed significantly (Fig 1I: Antibody x Age, F_4,30_ = 12.47, p<0.001). This was also the case for the detection of frank inclusions (Antibody x Age, F_4,31_ = 4.44, p<0.01).

At 8m of age MW8 was able to detect high levels of inclusions with post-hoc analysis determining that it was the most sensitive of the antibodies at this age (p<0.01). There were relative few cells that contained diffuse mHTT staining at either age. Despite the sensitivity of MW8, 1C2 failed to detect either diffuse staining or inclusions in 8m old animals and identified few inclusions in 18m old animals. Aside from MW8, only ubiquitin demonstrated high levels of staining in 8m old B6 HdhQ150 mice. As with the other mouse lines the levels of ubiquitin staining were constant over the 8m to 18m period. In the older 18m mice, post hoc analysis determined that MW8 remained the most sensitive of the antibodies for detecting frank inclusions demonstrating comparable levels across the two age groups (p<0.05), but interestingly S830 also detected much high levels of inclusions in the old mice (p<0.01) than it did at 8m despite the large numbers detected by MW8 in the younger mice, suggesting a change in conformation of the mHTT protein was required for the S830 binding at 18m of age.

The means and standard errors of the data sets are contained in Table 3 below. The table depicts how each antibody performs for each mouse line at the ages used and shows were antibodies failed in particular mouse lines.

**Table 3:** Means and standard errors of the data sets used in the present study. (See S1 File for raw data).

		Mean Number of Cells with Diffuse mHTT (no NIIs)				Mean Number of Cells with mHTT NII			
		Young		Old		Young		Old	
	Antibody	Mean	Std Error	Mean	Std Error	Mean	Std Error	Mean	Std Error
**R6/1**	S830	200750	39542	135071	45743	572564	112639	438681	49180
	MW8	0	-	0	-	524286	63345	530353	23636
	EM48	0	-	0	-	600399	45241	747742	47947
	1C2	204951	35059	0	-	0	-	99545	13300
	Ubiquitin	53715	8922	67425	16979	194368	33385	187625	15816
**YAC128**	S830	417324	39117	165299	35665	0	-	281218	41722
	MW8	169262	46895	0	-	17361	2053	528099	167620
	EM48	188560	33442	0	-	0	-	382846	37697
	1C2	156588	25003	268451	52096	0	-	97166	21196
	Ubiquitin	463573	38514	339775	75899	0	-	132443	15811
**HdhQ92**	S830	325042	27424	78398	13231	145545	20202	639032	68298
	MW8	277823	48395	0	-	0	-	370060	62949
	EM48	487886	14714	0	-	0	-	411493	43713
	1C2	153323	50856	0	-	0	-	299920	53162
	Ubiquitin	204037	49615	0	-	0	-	235407	40015
**HdhQ150**	S830	193762	29829	0	-	269899	46802	581021	43156
**(B6x129/Ola)**	MW8	198015	66316	0	-	201010	47904	569580	114339
	EM48	0	-	0	-	0	-	0	-
	1C2	0	-	0	-	316529	81570	494420	33892
	Ubiquitin	0	-	0	-	402699	65845	442095	44852
**HdhQ150**	S830	92378	29078	0	-	122834	40027	448266	22479
**(B6)**	MW8	0	-	0	-	729092	204290	638207	52992
	EM48	0	-	0	-	0	-	0	-
	1C2	0	-	0	-	0	-	190867	18399
	Ubiquitin	54969	15067	0	-	405961	80919	387533	80804

## Discussion

The results from the present study demonstrate clear differences in antibody effectiveness within the HD mouse lines. In the R61 mice S830, MW8 and EM48 were the most effective is detecting inclusion formations, with S830 and 1C2 also identifying cells with diffuse staining in the absence of an inclusion. In the YAC12 mice, diffuse staining of ubiquitin and MW8 were the most effective in 8m old mice. In the 18m old mice, MW8 was most sensitive in labelling mature inclusions but ubiquitin still had affinity for the diffuse staining. For the HdhQ92 model, there was a clear separation between early diffuse staining and late inclusion staining. EM48 was the most sensitive in the young animals with S830 detecting most inclusions at 18 months of age. In the original HdhQ150 mice, S830 was the most effective antibody overall, but MW8, 1C2 and ubiquitin were also effective at detecting frank inclusions. In the B6 HdhQ150 variant, MW8 was the most effective. As expected, the different mouse lines demonstrated different levels of sensitivity towards the different antibodies used. As a general rule, (and under the conditions employed in the present study) S830 and MW8 were the most effective of the antibodies, and1C2 the least (excepting the lack of EM48 binding in the HdhQ150 lines).

When the performance of each of the antibodies is considered in more general terms across the mouse lines it was clear that antibody selectivity for either form of mHTT was mouse line specific and was not generalizable across the strains, with the exception of S830 that demonstrated good levels of detection of mHTT in both diffuse and frank inclusion form, in all mouse lines relative to the other antibodies used. In contrast MW8 demonstrated most variability: exclusively selective for mature inclusions in the R6/1 and B6 HdhQ150 mice; comparable levels of detection for diffuse mHTT and inclusions at 8m but exclusive binding to mature inclusions at 18m in the original HdhQ150s; comparable detection of early diffuse staining and late inclusion staining in the HdhQ92s; and in the YAC128 mouse a modest level of detection of diffuse mHTT in 8m old mice that developed to be the most sensitive antibody in the 18m old mice. 1C2 binding was generally one of the weakest antibodies throughout suggesting that antibodies that target only the polyglutamine stretch of the mHTT may not be optimal. This was highlighted previously with MW family of antibodies in the R6/2 mouse [32], which were specific to particular cellular compartments. MW7 binding to the polyglutamine epitope was weak in HD mice that had clearly demonstrated NIIs with the polyP sensitive MW8 [32], suggesting that in some mouse lines antibodies directed at the polyglutamine stretch of the mHTT protein may not be the most sensitive for mHTT detection. In the present study, S830 and EM48 both of which were highly sensitive and bind to the polyglutamine stretch of the mHTT, also bind to a section of exon1 which likely aids mHTT detection. Of significant interest was that ubiquitin staining was remarkably stable across the age groups within each mouse line.

There has been a long-standing question regarding the role of ubiquitin and related proteolysis in the pathogenesis of HD [38–40] (reviewed in [51]). The evidence remains inconclusive as to whether the ubiquitination of mHTT is an early stage event, a late stage event or occurs concomitantly with aggregation development [26, 41, 42]. For the present study, it was not possible to explore the nature of ubiquitin staining in greater detail, but from our data we can see that in the R6/1 mice ubiquitin staining was relatively low at both ages suggesting that many inclusions available did not contain ubiquitin. In contrast ubiquitin binding in the YAC128 mice was particularly high in 8m old animals relative to the other antibodies used and this was in the absence of frank inclusion formations suggesting that in this mouse line, ubiquitin binding was an early event in the development of inclusion formations. However, in the HdhQ92 mouse diffuse and more punctate staining were to comparable levels with ubiquitin, again suggesting that ubiquitin is present in both diffuse mHTT and later inclusions. In both HdhQ150 lines concentrated ubiquitin staining was found at both ages as comparable levels. The variation across the mouse lines in ubiquitin binding suggests that generalisations between models are not informative. Taking our data as a whole is clear that ubiquitin binding of mHTT can be present during the early stages of inclusion development and not simply a late stage event as previously suggested [26, 52]. In agreement with these studies, our data suggests that not all inclusions are ubiquitinated [26, 52]. Further hypothesising about the role of ubiquitin in the development of inclusion bodies is beyond the scope of the present report.

Simply using different background strains produced marked effects on the binding profiles of the antibodies. Whilst overall levels of pathology were similar in the 18m old HdhQ150 mice of the two lines with S830 and MW8 demonstrating good binding, levels of 1C2 binding was particularly poor in the pure B6 line again demonstrating that even in mouse lines that carry the same construct but on different backgrounds, antibody sensitivity may vary. In the present study, this variation in 1C2 binding occurred even when the two mouse lines demonstrated comparable asymptotic sensitivity with other antibodies.

## Conclusions

The data from the present study highlights the variation in antibody binding that exists within the HD mouse lines and suggests that generalisations between studies using different antibodies within the same line should be made with caution. Similarly, generalisation across mouse lines with the same antibody may not be advisable. With reference to the role of ubiquitin in the development of inclusion bodies, the present study demonstrates the ubiquitin binding was remarkably consistent across age groups regardless of HD mouse model or mHTT aggregation stage even when binding of the other antibodies increased with age. Our data suggest that ubiquitin binding is not present in all inclusion bodies or necessary for their development.

## Supporting Information

S1 File Last Inclusion count data (S830 with CA). (XLSX)

## References

[1] The Huntington's Disease Collaborative Research G. A novel gene containing a trinucleotide repeat that is expanded and unstable on Huntington's disease chromosomes. Cell. 1993;72(6):971–83. 8458085 10.1016/0092-8674(93)90585-e

[2] Vonsattel JP, Myers RH, Stevens TJ, Ferrante RJ, Bird ED, Richardson EP Jr. Neuropathological classification of Huntington's disease. JNeuropatholExpNeurol. 1985;44(6):559–77. 10.1097/00005072-198511000-00003 2932539

[3] Sapp E, Schwarz C, Chase K, Bhide PG, Young AB, Penney J, et al Huntingtin localization in brains of normal and Huntington's disease patients. AnnNeurol. 1997;42(4):604–12. 10.1002/ana.410420411 9382472

[4] Maat-Schieman ML, Dorsman JC, Smoor MA, Siesling S, Van Duinen SG, Verschuuren JJ, et al Distribution of inclusions in neuronal nuclei and dystrophic neurites in Huntington disease brain. JNeuropatholExpNeurol. 1999;58(2):129–37. 10.1097/00005072-199902000-00003 10029096

[5] Herndon ES, Hladik CL, Shang P, Burns DK, Raisanen J, White CL 3rd. Neuroanatomic profile of polyglutamine immunoreactivity in Huntington disease brains. Journal of neuropathology and experimental neurology. 2009;68(3):250–61. 10.1097/NEN.0b013e318198d320 19225411 PMC2756075

[6] Gutekunst CA, Li SH, Yi H, Mulroy JS, Kuemmerle S, Jones R, et al Nuclear and neuropil aggregates in Huntington's disease: relationship to neuropathology. J Neurosci. 1999;19(7):2522–34. 10087066 10.1523/JNEUROSCI.19-07-02522.1999 PMC6786077

[7] Gourfinkel-An I, Cancel G, Duyckaerts C, Faucheux B, Hauw JJ, Trottier Y, et al Neuronal distribution of intranuclear inclusions in Huntington's disease with adult onset. Neuroreport. 1998;9(8):1823–6. 9665608 10.1097/00001756-199806010-00028

[8] DiFiglia M, Sapp E, Chase KO, Davies SW, Bates GP, Vonsattel JP, et al Aggregation of huntingtin in neuronal intranuclear inclusions and dystrophic neurites in brain. Science. 1997;277(5334):1990–3. 9302293 10.1126/science.277.5334.1990

[9] Wheeler VC, White JK, Gutekunst CA, Vrbanac V, Weaver M, Li XJ, et al Long glutamine tracts cause nuclear localization of a novel form of huntingtin in medium spiny striatal neurons in HdhQ92 and HdhQ111 knock-in mice. HumMolGenet. 2000;9(4):503–13. 10.1093/hmg/9.4.503 10699173

[10] von Horsten S, Schmitt I, Nguyen HP, Holzmann C, Schmidt T, Walther T, et al Transgenic rat model of Huntington's disease. HumMolGenet. 2003;12(6):617–24. 10.1093/hmg/ddg075 12620967

[11] Slow EJ, van Raamsdonk J, Rogers D, Coleman SH, Graham RK, Deng Y, et al Selective striatal neuronal loss in a YAC128 mouse model of Huntington disease. HumMolGenet. 2003;12(13):1555–67. 10.1093/hmg/ddg169 12812983

[12] Slow EJ, Graham RK, Osmand AP, Devon RS, Lu G, Deng Y, et al Absence of behavioral abnormalities and neurodegeneration in vivo despite widespread neuronal huntingtin inclusions. ProcNatlAcadSciUSA. 2005;102(32):11402–7. 10.1073/pnas.0503634102 PMC1183566 16076956

[13] Schilling G, Becher MW, Sharp AH, Jinnah HA, Duan K, Kotzuk JA, et al Intranuclear inclusions and neuritic aggregates in transgenic mice expressing a mutant N-terminal fragment of huntingtin. HumMolGenet. 1999;8(3):397–407. 10.1093/hmg/8.3.397 9949199

[14] Scherzinger E, Lurz R, Turmaine M, Mangiarini L, Hollenbach B, Hasenbank R, et al Huntingtin-encoded polyglutamine expansions form amyloid-like protein aggregates in vitro and in vivo. Cell. 1997;90(3):549–58. 9267034 10.1016/s0092-8674(00)80514-0

[15] Mangiarini L, Sathasivam K, Seller M, Cozens B, Harper A, Hetherington C, et al Exon 1 of the HD gene with an expanded CAG repeat is sufficient to cause a progressive neurological phenotype in transgenic mice. Cell. 1996;87(3):493–506. 8898202 10.1016/s0092-8674(00)81369-0

[16] Mangiarini L, Sathasivam K, Mahal A, Mott R, Seller M, Bates GP. Instability of highly expanded CAG repeats in mice transgenic for the Huntington's disease mutation. NatGenet. 1997;15(2):197–200. 10.1038/ng0297-197 9020849

[17] Lin CH, Tallaksen-Greene S, Chien WM, Cearley JA, Jackson WS, Crouse AB, et al Neurological abnormalities in a knock-in mouse model of Huntington's disease. HumMolGenet. 2001;10(2):137–44. 10.1093/hmg/10.2.137 11152661

[18] Sanchez I, Mahlke C, Yuan J. Pivotal role of oligomerization in expanded polyglutamine neurodegenerative disorders. Nature. 2003;421(6921):373–9. 12540902 10.1038/nature01301

[19] Rubinsztein DC, Wyttenbach A, Rankin J. Intracellular inclusions, pathological markers in diseases caused by expanded polyglutamine tracts? JMedGenet. 1999;36(4):265–70. PMC1734357 10227391

[20] Rubinsztein DC. The roles of intracellular protein-degradation pathways in neurodegeneration. Nature. 2006;443(7113):780–6. 17051204 10.1038/nature05291

[21] Ordway JM, Detloff PJ. In vitro synthesis and cloning of long CAG repeats. Biotechniques. 1996;21(4):609–10, 12 8891208 10.2144/96214bm08

[22] Saudou F, Finkbeiner S, Devys D, Greenberg ME. Huntingtin acts in the nucleus to induce apoptosis but death does not correlate with the formation of intranuclear inclusions. Cell. 1998;95(1):55–66. 9778247 10.1016/s0092-8674(00)81782-1

[23] Morton AJ, Lagan MA, Skepper JN, Dunnett SB. Progressive formation of inclusions in the striatum and hippocampus of mice transgenic for the human Huntington's disease mutation. J Neurocytol. 2000;29(9):679–702. 11353291 10.1023/a:1010887421592

[24] Morton AJ, Glynn D, Leavens W, Zheng Z, Faull RL, Skepper JN, et al Paradoxical delay in the onset of disease caused by super-long CAG repeat expansions in R6/2 mice. NeurobiolDis. 2009;33(3):331–41. 10.1016/j.nbd.2008.11.015 19130884

[25] Hansson O, Guatteo E, Mercuri NB, Bernardi G, Li XJ, Castilho RF, et al Resistance to NMDA toxicity correlates with appearance of nuclear inclusions, behavioural deficits and changes in calcium homeostasis in mice transgenic for exon 1 of the huntington gene. EurJ Neurosci. 2001;14(9):1492–504. 11722611 10.1046/j.0953-816x.2001.01767.x

[26] Gong B, Kielar C, Morton AJ. Temporal separation of aggregation and ubiquitination during early inclusion formation in transgenic mice carrying the Huntington's disease mutation. PLoSOne. 2012;7(7):e41450. 10.1371/journal.pone.0041450 PMC3404089 22848498

[27] Bodner RA, Housman DE, Kazantsev AG. New directions for neurodegenerative disease therapy: using chemical compounds to boost the formation of mutant protein inclusions. Cell cycle (Georgetown, Tex). 2006;5(14):1477–80. 10.4161/cc.5.14.2929 16861893

[28] Arrasate M, Mitra S, Schweitzer ES, Segal MR, Finkbeiner S. Inclusion body formation reduces levels of mutant huntingtin and the risk of neuronal death. Nature. 2004;431(7010):805–10. 15483602 10.1038/nature02998

[29] Abada YS, Nguyen HP, Ellenbroek B, Schreiber R. Reversal learning and associative memory impairments in a BACHD rat model for Huntington disease. PloS one. 2013;8(11):e71633 10.1371/journal.pone.0071633 24223692 PMC3815226

[30] Miller JA, Cai C, Langfelder P, Geschwind DH, Kurian SM, Salomon DR, et al Strategies for aggregating gene expression data: the collapseRows R function. BMC bioinformatics. 2011;12:322 10.1186/1471-2105-12-322 21816037 PMC3166942

[31] Trottier Y, Lutz Y, Stevanin G, Imbert G, Devys D, Cancel G, et al Polyglutamine expansion as a pathological epitope in Huntington's disease and four dominant cerebellar ataxias. Nature. 1995;378(6555):403–6. 7477379 10.1038/378403a0

[32] Milnerwood AJ, Cummings DM, Dallerac GM, Brown JY, Vatsavayai SC, Hirst MC, et al Early development of aberrant synaptic plasticity in a mouse model of Huntington's disease. HumMolGenet. 2006;15(10):1690–703. 10.1093/hmg/ddl092 16600988

[33] Li H, Li SH, Cheng AL, Mangiarini L, Bates GP, Li XJ. Ultrastructural localization and progressive formation of neuropil aggregates in Huntington's disease transgenic mice. HumMolGenet. 1999;8(7):1227–36. 10.1093/hmg/8.7.1227 10369868

[34] Ko J, Ou S, Patterson PH. New anti-huntingtin monoclonal antibodies: implications for huntingtin conformation and its binding proteins. Brain ResBull. 2001;56(3–4):319–29. 10.1016/s0361-9230(01)00599-8 11719267

[35] Landles C, Sathasivam K, Weiss A, Woodman B, Moffitt H, Finkbeiner S, et al Proteolysis of mutant huntingtin produces an exon 1 fragment that accumulates as an aggregated protein in neuronal nuclei in Huntington disease. J BiolChem. 2010;285(12):8808–23. 10.1074/jbc.M109.075028 PMC2838303 20086007

[36] Ciechanover A, Stanhill A. The complexity of recognition of ubiquitinated substrates by the 26S proteasome. Biochimica et biophysica acta. 2014;1843(1):86–96. 10.1016/j.bbamcr.2013.07.007 23872423

[37] Ciechanover A. The ubiquitin-proteasome proteolytic pathway. Cell. 1994;79(1):13–21. 7923371 10.1016/0092-8674(94)90396-4

[38] Waelter S, Boeddrich A, Lurz R, Scherzinger E, Lueder G, Lehrach H, et al Accumulation of mutant huntingtin fragments in aggresome-like inclusion bodies as a result of insufficient protein degradation. MolBiolCell. 2001;12(5):1393–407. 10.1091/mbc.12.5.1393 PMC34592 11359930

[39] Venkatraman P, Wetzel R, Tanaka M, Nukina N, Goldberg AL. Eukaryotic proteasomes cannot digest polyglutamine sequences and release them during degradation of polyglutamine-containing proteins. Molecular cell. 2004;14(1):95–104. 15068806 10.1016/s1097-2765(04)00151-0

[40] Bence NF, Sampat RM, Kopito RR. Impairment of the ubiquitin-proteasome system by protein aggregation. Science. 2001;292(5521):1552–5. 11375494 10.1126/science.292.5521.1552

[41] Maynard CJ, Bottcher C, Ortega Z, Smith R, Florea BI, Diaz-Hernandez M, et al Accumulation of ubiquitin conjugates in a polyglutamine disease model occurs without global ubiquitin/proteasome system impairment. Proceedings of the National Academy of Sciences of the United States of America. 2009;106(33):13986–91. 10.1073/pnas.0906463106 19666572 PMC2729007

[42] Meade CA, Deng YP, Fusco FR, Del Mar N, Hersch S, Goldowitz D, et al Cellular localization and development of neuronal intranuclear inclusions in striatal and cortical neurons in R6/2 transgenic mice. The Journal of comparative neurology. 2002;449(3):241–69. 12115678 10.1002/cne.10295

[43] Her LS, Lin JY, Fu MH, Chang YF, Li CL, Tang TY, et al The Differential Profiling of Ubiquitin-Proteasome and Autophagy Systems in Different Tissues before the Onset of Huntington's Disease Models. Brain pathology. 2014. 10.1111/bpa.12191 PMC8029242 25178567

[44] Bayram-Weston Z, Jones L, Dunnett SB, Brooks SP. Light and electron microscopic characterization of the evolution of cellular pathology in the R6/1 Huntington's disease transgenic mice. Brain ResBull. 2012;88(2–3):104–12. 10.1016/j.brainresbull.2011.07.009 21801812

[45] Bayram-Weston Z, Jones L, Dunnett SB, Brooks SP. Light and electron microscopic characterization of the evolution of cellular pathology in YAC128 Huntington's disease transgenic mice. Brain ResBull. 2012;88(2–3):137–47. 10.1016/j.brainresbull.2011.05.005 21620935

[46] Bayram-Weston Z, Jones L, Dunnett SB, Brooks SP. Light and electron microscopic characterization of the evolution of cellular pathology in HdhQ92 Huntington's disease knock-in mice. Brain ResBull. 2012;88(2–3):171–81. 10.1016/j.brainresbull.2011.03.013 21513775

[47] Bayram-Weston Z, Torres EM, Jones L, Dunnett SB, Brooks SP. Light and electron microscopic characterization of the evolution of cellular pathology in the Hdh(CAG)150 Huntington's disease knock-in mouse. Brain ResBull. 2012;88(2–3):189–98. 10.1016/j.brainresbull.2011.03.014 21511013

[48] Wheeler VC, Auerbach W, White JK, Srinidhi J, Auerbach A, Ryan A, et al Length-dependent gametic CAG repeat instability in the Huntington's disease knock-in mouse. HumMolGenet. 1999;8(1):115–22. 10.1093/hmg/8.1.115 9887339

[49] Sieradzan KA, Mechan AO, Jones L, Wanker EE, Nukina N, Mann DM. Huntington's disease intranuclear inclusions contain truncated, ubiquitinated huntingtin protein. Experimental neurology. 1999;156(1):92–9. 10192780 10.1006/exnr.1998.7005

[50] Abercrombie M. Estimation of nuclear population from microtome sections. AnatRec. 1946;94:239–47. 10.1002/ar.1090940210 21015608

[51] Ortega Z, Lucas JJ. Ubiquitin-proteasome system involvement in Huntington's disease. Frontiers in molecular neuroscience. 2014;7:77 10.3389/fnmol.2014.00077 25324717 PMC4179678

[52] Senut MC, Suhr ST, Kaspar B, Gage FH. Intraneuronal aggregate formation and cell death after viral expression of expanded polyglutamine tracts in the adult rat brain. JNeurosci. 2000;20(1):219–29. 10627599 10.1523/JNEUROSCI.20-01-00219.2000 PMC6774116

